# Dataset concerning the analytical approximation of the *Ae_3_* temperature

**DOI:** 10.1016/j.dib.2016.11.073

**Published:** 2016-12-09

**Authors:** B.L. Ennis, E. Jimenez-Melero, R. Mostert, B. Santillana, P.D. Lee

**Affiliations:** a Tata Steel Research and Development, 1970 CA IJmuiden, The Netherlands; b The School of Materials, University of Manchester, Oxford Road, M13 9PL Manchester, UK; c Dalton Cumbrian Facility, Westlakes Science and Technology Park, CA24 3HA Moor Row, UK; d Manchester X-Ray Imaging Facility, Research Complex at Harwell, RAL, OX11 0FA Didcot, UK

**Keywords:** Steel, Phase transformation temperature, *Ae3*, Approximation method

## Abstract

In this paper we present a new polynomial function for calculating the local phase transformation temperature (*Ae*_*3*_) between the austenite+ferrite and the fully austenitic phase fields during heating and cooling of steel:

$$Ae_{3}({}^{\circ}C)=c_{0}+\sum_{X,k}c_{Xk}X^{k}+\sum_{X,Y,k,m}c_{XkYm}X^{k}Y^{m}+\sum_{X,Y,Z,k,m,n}c_{XkYmZn}X^{k}Y^{m}Z^{n}$$

The dataset includes the terms of the function and the values for the polynomial coefficients for major alloying elements in steel. A short description of the approximation method used to derive and validate the coefficients has also been included. For discussion and application of this model, please refer to the full length article entitled “The role of aluminium in chemical and phase segregation in a TRIP-assisted dual phase steel” 10.1016/j.actamat.2016.05.046 (Ennis et al., 2016) [1].

**Specifications Table**

**Table:** 

Subject area	*Steel metallurgy*
More specific subject area	*Phase transformations*
Type of data	*Tables and equations*
How data was acquired	*The approximation of the Ae*_*3*_*temperature was constructed in two steps: in the first step a large number of compositions with the associated Ae*_*3*_*temperatures were generated; this was followed by multiple regression to find a suitable approximation*
Data format	*Analysed – Contributions to polynomial coefficients in carbon para-equilibrium equation*
Experimental factors	*Numerical analysis was carried out on model alloys generated from MTDATA*[2]*and resulted in the polynomial function, which is described in more detail in this paper.*
Experimental features	*The approximation of the Ae*_*3*_*temperature was constructed in two steps: in the first step a large number of compositions with the associated Ae3 temperatures were generated; this was followed by multiple regression to find a suitable approximation*
Data source location	*N/A*
Data accessibility	*Data is within this article.*

**Value of the data**

• Improved polynomial relationship of phase transformation temperature for major alloying elements in steel.
• Can be directly used to compute phase transformation temperature for any alloy within the computed range.
• Compares well with full thermodynamic model data, but with simple polynomial function.
• This function can be seen as an extension of the Andrews expression [3], see Eq. (1), to include the role of carbon and aluminium on critical transformation temperature:
  (1) $$Ae_{3}\;({}^{\circ}C)\;=\;\text{910}\;-25C_{Mn}+\mathrm{60}C_{Si}\;-11C_{Cr}$$
• Where *Ae*_*3*_ temperature is expressed in °C and concentrations in wt. %.

## 1 Data

There are three tables used to describe the numerical approximation of the *Ae*_*3*_ temperature:

Table 1 gives the maximum valid composition range based on the model alloys used.

Table 2 lists the contribution of each element to the polynomial coefficients in the derived function given in Eq. (6) in Ref. [1]:

(2) $$Ae_{3}({}^{\circ}C)=c_{0}+\sum_{X,k}c_{Xk}X^{k}+\sum_{X,Y,k,m}c_{XkYm}X^{k}Y^{m}+\sum_{X,Y,Z,k,m,n}c_{XkYmZn}X^{k}Y^{m}Z^{n}$$

where *Ae*_*3*_ temperature is expressed in °C and concentrations in wt. %. Under para-equilibrium conditions carbon is the only chemical element that changes its concentration during transformation and to avoid repetitive calculations it is advantageous to write *Ae*_3_ as a polynomial in carbon, [C], as follows:

(3) $$Ae_{3}=\sum_{i}c_{i}^{*}{\left[C\right]}^{i}$$

The relationships of $c_{i}^{*}$ to the constants, *c*, are listed in Table 3.

## 2 Experimental design, materials and methods

The approximation of the *Ae*_3_ temperature was constructed in two steps: in the first step a large number of compositions with the associated *Ae*_3_ temperatures were generated; this was followed by multiple regression to find a suitable approximation. For each run, a total of 100,000 *Ae*_3_ temperatures were generated with [C]<0.8 wt. % and within the range of validity for all other chemical elements given in Table 1. The value of each chemical element was chosen independently of all the other elements and was taken from a uniform distribution between 0 and the maximum allowed content. The SAS procedure ‘reg’ with the option ‘selection=stepwise’ chose terms from a large bank that contributed significantly to *Ae*_3_. Terms that did not improve the fit to the data were not included. The bank of terms consisted of:

Chemical elements.

Chemical elements squared.

[C], Mn, Si and Al to the third power.

[C]^4^, [C]^5^, and [C]^6^.

The product of [C], Mn, Al and Si with all other elements.

The product of [C]^2^, [C]^3^, [C]^4^, Mn^2^, Al^2^, and Si^2^ with the other elements.

[C]MnCr.

Since the starting temperature for the model is 910 °C, all calculated *Ae*_3_ temperatures higher than this value are assigned the starting value. Calculated *Ae*_3_ temperatures higher than 910 °C should be approached with caution, because some extrapolation will have taken place. This is especially true for Al and Si compositions at the upper end of the valid range.

A measure of success of the approximation is the difference between the full MTDATA expressions and the values obtained from the approximation. The standard deviation of the approximation is 4.9 °C, which is much smaller than the undercooling at which nucleation is assumed to take place, with an offset of 0.0 °C. The fit for *Ae*_3_ was also determined for a second, independent set of 100,000 *Ae*_3_ temperatures. The differences between the two sets were small; the differences with Andrews׳ expression, Eq. (1) are somewhat larger, with an average difference of 11 °C and a standard deviation of 22 °C.

## Figures and Tables

**Table 1:** Maximum valid compositions (wt. %) and calculated value of *Ae*_*3*_ from the approximation.

[C]	Mn	Cr	Si	Al	Maximum calculated *Ae*_*3*_
0.8	2.5	1.0	1.5	2.0	910 °C

**Table 2:** Contributions to polynomial coefficients in carbon para-equilibrium equation.

Contributes to	Product of elements	Constant	Units
$c_{0}^{*}$	[intercept]	918.6	°C
	Al	161.4	°C/wt. %
	Cr	−9.4	
	Mn	−57.1	
	Si	50.2	
	AlCr	−4.2	°C/(wt. %)^2^
	AlMn	−18.2	
	AlSi	16.0	
	CrMn	−3.6	
	MnSi	−1.9	
	Al^2^	19.4	
	Cr^2^	1.1	
	Mn^2^	1.5	
	Si^2^	5.0	
	Al^3^	−0.9	°C/(wt. %)^3^
	Mn^3^	0.4	
	Al^2^Cr	1.1	
	Al^2^Mn	3.5	
	Al^2^Si	−1.2	
	Mn^2^Cr	0.8	
	Mn^2^Si	−0.5	
			
$c_{1}^{*}$	[C]	−720.0	°C/wt. %
	[C]Al	−380.2	°C/(wt. %)^2^
	[C]Cr	−12.4	
	[C]Mn	108.6	
	[C]Si	−122.1	
	[C]MnCr	9.7	°C/(wt. %)^3^
	[C]Al^2^	−11.3	
	[C]Si^2^	−5.9	
			
$c_{2}^{*}$	[C]^2^	1608.4	°C/(wt. %)^2^
	[C]^2^Al	399.9	°C/(wt. %)^3^
	[C]^2^Mn	−212.4	
	[C]^2^Si	71.4	
			
$c_{3}^{*}$	[C]^3^	−2981.2	°C/(wt. %)^3^
	[C]^3^Al	−188.1	°C/(wt. %)^4^
	[C]^3^Mn	259.7	
			
$c_{4}^{*}$	[C]^4^	4051.0	°C/(wt. %)^4^
	[C]^4^Cr	17.3	°C/(wt. %)^5^
	[C]^4^Mn	−94.5	
$c_{5}^{*}$	[C]^5^	−3388.1	°C/(wt. %)^5^
$c_{6}^{*}$	[C]^6^	1227.8	°C/(wt. %)^6^

**Table 3:** Relationship of $c_{i}^{*}$ terms in the carbon polynomial in Eq.(3) to the constants, *c*_*i*_.

$c_{0}^{*}=c_{0}+\sum_{\underset{X\neq [C]}{X,k}}c_{Xk}X^{k}+\sum_{\underset{X,Y\neq [C]}{X,Y,k}}c_{XkYm}X^{k}Y$
$c_{1}^{*}=c_{C,1}[C\;]+\sum_{\underset{X\neq [C]}{X,k}}c_{Xk[C1]}X^{k}[C\;]+c_{Mn1Cr1[C1]}MnCr[C\;]$
$c_{2}^{*}=c_{C,2}{\left[C\;\right]}^{2}+\sum_{X_{X\neq [C]}}c_{X1[C2]}X{\left[C\;\right]}^{2}$
$c_{3}^{*}=c_{C,3}{\left[C\;\right]}^{3}+\sum_{X_{X\neq [C]}}c_{X1[C3]}X{\left[C\;\right]}^{3}$
$c_{4}^{*}=c_{C,4}{\left[C\;\right]}^{4}+\sum_{X_{X\neq [C]}}c_{X1[C4]}X{\left[C\;\right]}^{4}$
$c_{5}^{*}=c_{C,5}{\left[C\;\right]}^{5}$
$c_{6}^{*}=c_{C,6}{\left[C\;\right]}^{6}$
